# The Silent Threat in Women’s Health: Work and Family Conflict

**DOI:** 10.1089/whr.2024.0088

**Published:** 2024-09-11

**Authors:** Sevil Cıcek Ozdemır, Selmin Senol

**Affiliations:** Faculty of Health Sciences, Nursing Department, Kutahya Health Sciences University, Kutahya, Turkey.

**Keywords:** women, health, female, family, work, conflict

## Abstract

**Aim:**

This study aimed to examine the level of work–family conflict and the factors affecting it among women academic and administrative staff working at the university.

**Materials and Methods:**

The study is descriptive and cross-sectional. We collected data online from women academic and administrative staff at two different universities from December 2022 to June 2023. The data for the study were collected by using the Personal Information Form, Work–Family Conflict Scale, and Family–Work Conflict Scale.

**Results:**

The mean Work–Family and Family–Work Conflict Scale scores of the female university staff participating in the study were 15.40 ± 5.02 and 12.02 ± 4.76, respectively. Academic staff were found to have higher mean scores in the Work–Family Conflict Scale (*U* = 1942.5, *p* = 0.034) and the Family–Work Conflict Scale (*U* = 1972, *p* = 0.042) than administrative staff. The Work–Family Conflict Scale score was affected by the status of sharing domestic responsibilities with the spouse (χ^2^ = 8.855, *p* = 0.012). Likewise, it was found that the Family–Work Conflict Scale score was affected by the presence of elderly and/or disabled individuals who were in need of help and support at home (*U* = 772, *p* = 0.030), family type (χ^2^ = 8.013, *p* = 0.018), and having children (*U* = 2917, *p* = 0.028). There was a significant negative correlation between the Work–Family Conflict Scale and daily time allocated to oneself and their spouse (*r* = −0.163, *p* = 0.034; *r* = −0.189, *p* = 0.013), and a significant positive correlation between the Work–Family Conflict Scale and number of children (*r* = 0.185, *p* = 0.04), age of children (*r* = 0.204, *p* = 0.03), and daily time allocated to child/children (*r* = 0.250, *p* = 0.001).

**Conclusions:**

Work–family conflict is affected by variables related to the work and family roles of university staff.

## Introduction

Work and family are at the center of human life. The roles and responsibilities required by these two areas constitute an important part of individuals’ lives. Yet, work–family conflict is experienced as a result of conflicting demands from work and family at the same time. Work–family conflict is a type of conflict arising when role and responsibility pressures from the work and family domains become mutually incompatible and contradictory between work and family.^1^ Although work–family conflict is compelling for women and men, it is reported that it has greater effects on women and conflict management is more difficult for women.^2^ Apart from assuming primary roles in family responsibilities (housework, etc.) and care (childcare, elderly care, etc.), women also take an active role in business life.^3,4^ Many women experience cross-domain conflict in trying to simultaneously meet work and family demands and responsibilities.^5,6^

University administrative staff face a heavy workload in administrative work related to both staff and students.^7^ Meanwhile, academic staff are expected to constantly renew and improve themselves, conduct scientific research, and publish their results in high-impact journals. They also have job responsibilities that require a lot of time and energy, such as teaching, consulting, editing, and committee membership. Effective time management and balancing work and family demands with multiple job responsibilities are major concerns for female university staff, especially academic staff.^8^ The increasing trend in the number of women academic staff, which is observed worldwide, has also been observed in Turkey in recent years. When this trend is taken into consideration, work–family conflict phenomena are also increasing.^6^ Currently, while women academicians try to fulfill the requirements of their profession, they are also obliged to do housework, childcare, and similar tasks. Women academicians have to meet the demands associated with many roles such as mother, wife, researcher, and educator.^9,10^

Work–family conflict affects individuals’ psychological health with such aspects as stress, burnout, emotional exhaustion, and depersonalization.^11^ Therefore, women face problems such as anxiety, depression, disharmony, role tension and dissatisfaction, feeling lonely and restless, guilt, and a lack of time for themselves. In addition, caregiving roles in the family, such as housework and childcare, increase stress in women. While these problems are a source of stress for women in the world of education, career, and working life,^12^ it also affects family processes with a reduction in women’s participation in family care and commitment, a decrease in marital satisfaction, problems in children’s behavioral, developmental, and relational structures, and deterioration in communication and relationships with parents and the social environment.^13^ These problems provoke situations such as stress and tension in work life, deterioration in attendance and concentration, job quitting, decreased job and role satisfaction, decreased motivation and performance, inefficiency in teamwork and burnout, and reduced life satisfaction.^14^ Work–family conflict has a silent and serious potential to threaten women’s health beyond creating a negative impact on family and work.^15^ Although there are examples of literature examining work–family conflict, study results are contradictory.^10,16–18^ This study aimed to examine the level of work–family conflict and the factors affecting it among women academic and administrative staff.

## Materials and Methods

### Type of study

Descriptive and cross-sectional methods were used in the study.

### Place of the study

The research was conducted at two different universities (A and B) in a province in the Aegean Region of Turkey. The data were collected online using an online survey system through Google Forms.

### Population and sample of the study

Women academic and administrative staff working in two different universities in a province in the Aegean Region constituted the population of the study. Sampling was not taken into account in the study; a total of 170 academic and administrative staff, who completed the data collection form totally, were included in the study. Academic or administrative staff working in one of the two universities in a province in the Aegean Region, who are women and who volunteered to participate in the study, were included in the study.

### Data collection tools

The data for the study were collected by using the personal information form, Work–Family Conflict Scale, and Family–Work Conflict Scale prepared by the researchers based on the literature review.

#### Personal information form

It consists of a total of 25 questions to determine the sociodemographic characteristics of academic and administrative staff and the characteristics related to work and family life.

#### Work–family conflict scale and family–work conflict scale

The level of work–family conflict among university staff was measured using the Work–Family Conflict Scale and the Family–Work Conflict Scale. The scale developed by Netemeyer et al. was adapted into Turkish by Efeoğlu.^19,20^ The scale, which aims to measure the levels of Work–Family Conflict Scale arising from work and Family–Work Conflict Scale arising from family, consists of two subdimensions. Work-to-family conflict emerges when experiences at work affect family life, while family-to-work conflict emerges when family responsibilities interfere with work duties. The Work–Family Conflict Scale and the Family–Work Conflict Scale consist of 10 items. In the study, a 5-point Likert scale was used: (1) Strongly Disagree, (2) Disagree, (3) Undecided, (4) Agree, and (5) Strongly Agree. The higher the score obtained from the scales, the higher the level of conflict. Based on the reliability analyses conducted by Netemeyer et al., Cronbach’s alpha reliability coefficients were found to be 0.88 and 0.89, respectively. According to the results of the reliability analysis conducted by Efeoğlu, the alpha value for the whole scale is 0.82, 0.88 for Work–Family Conflict Scale, and 0.85 for Family–Work Conflict Scale. In this study, Cronbach’s alpha reliability coefficient is 0.902 and 0.913, respectively.

### Research implementation

The data were collected online from December 2022 to June 2023. The questions in the data collection form were converted into an online survey using Google Forms. The online survey link was sent to the university staff’s email account with the university extension, and the university staff was invited to the research. In the first step, the email included explanatory information about the research and a voluntary consent form. In the second step, the online survey link was presented to those who agreed to participate in the study. The volunteers who wanted to participate in the study clicked on the online link and filled out the data collection form. Participants marked the most appropriate option for themselves to the questions in the data collection form.

### Data analyses

The research data were analyzed using SPSS 23.0 (Statistical Package for Social Sciences, version 23.0, for Windows). Number, percentage, mean, and standard deviation descriptive statistics were used to analyze the data. In addition, in the determination of the factors affecting the work–family conflict levels of university staff, the Mann–Whitney *U* test (Z-table value) was used to compare the measurement values of two independent groups for data that do not have a normal distribution; Kruskall–Wallis H test (χ^2^-table value) statistics were used to compare three or more independent groups. For significant variables, Dunn–Bonferroni pairwise comparison test was used to determine which group was responsible for the difference. Spearman correlation analysis was also applied. The significance was accepted as *p* < 0.05.

### Ethical aspects of the study

Ethics Committee Permission was obtained from the Non-Interventional Clinical Research Ethics Committee (date and number: 22.09.2022–64660) of the university where the study was conducted. Then, written institutional permission was obtained from both universities where the study was conducted. The university staff were informed about the research via email before the data collection process, and their consent for participation was sought. Those who stated that they volunteered to participate in the study participated in the study by clicking on the online link.

## Results

The mean age of the university staff participating in the study was 36.85 ± 7.21; 60.6% of them had an income equal to their expenses. The majority of the staff (90.6%) have nuclear family type, 5.9% have extended family type, and 3.5% live alone. In terms of educational status, 47.1% have completed their doctorate and 32.4% have completed their master’s degree. Among the spouses, 20.1% have completed their doctorate, 27.8% have completed their master’s degree, and 41.7% have a bachelor’s degree. Around 67.6% of the staff are married and 32.4% are single. When the duration of marriage is examined, 39.1% have been married for 11 years or more, 19.1% for 8–10 years, 23.5% for 4–7 years, and 18.3% for 1–3 years. Of the university staff participating in the study, 57.6% were working at University A, 42.4% at University B, and 39.4% had a working period of 11 years or more. Of the university staff, 77.6% were academic staff and 22.4% were administrative staff. When the academic titles of university staff are examined, 3.8% were professor, 15.2% were associate professor, 31.1% assistant professor, 4.5% lecturer, PhD, 4.5% research assistant, PhD, 23.5% lecturer, and 17.4% research assistant. Approximately, 67.4% academic staff have no administrative positions, and the average weekly course load is 11.7 ± 11.98. The average weekly time allocated by the university staff to work at home, daily housework at home, daily for self, daily for spouse, and daily for child/children was 7.74 ± 9.03, 3.05 ± 4.37, 1.5 ± 1.82, 1.15 ± 1.31, and 1.5 ± 1.86, respectively. Approximately, 8.8% of the university staff have elderly and/or disabled individuals living who need help and support at home. Regarding housework and childcare at home, 52.2% of the staff receive help from family members and 47.8% from paid caregivers. The percentages of university staff who share domestic responsibilities with their spouses every day in the evening after work, on weekends, and not sharing domestic responsibilities are 53.9%, 39.1%, and 7%, respectively. In addition, the rates of sharing childcare responsibilities with their spouses every day in the evening after work, sharing them on weekends, and not sharing them are 70.9%, 14%, and 15%, respectively (Table 1).

**Table 1.** Demographic and Working Life Characteristics of University Staff

Characteristics	*n*	%
Age (Mean ± SD) (Min, Max)	36.85 ± 7.21 (25, 61)	
Income status		
Low (income less than expenditure)	14	8.2
Medium (income equal to expenditure)	103	60.6
High (income more than expenditure)	53	31.2
Family type		
Nuclear family	154	90.6
Extended family	10	5.9
I live alone	6	3.5
Educational status		
High school graduate	7	4
Bachelor’s degree	28	16.5
Master’s degree	55	32.4
Doctorate degree	80	47.1
Marital status		
Single	55	32.4
Married	115	67.6
Duration of marriage / year (*n* = 115)		
1–3 years	21	18.3
4–7 years	27	23.5
8–10 years	22	19.1
11 years or more	45	39.1
Age of spouse (Mean ± SD) (*n* = 115) (Min, Max)	39.98 ± 8 (25, 74)	
Spouse’s education status (*n* = 115)		
High school graduate	12	10.4
Bachelor’s degree	48	41.7
Master’s degree	32	27.8
Doctorate degree	23	20.1
Number of children (Mean ± SD)	1.15 ± 0.84	
Ages of children (Mean ± SD)	10.37 ± 11.7	
Institution of working		
A	98	57.6
B	72	42.4
Position		
Administrative staff	38	22.4
Academic staff	132	77.6
Academic title (*n* = 132)		
Prof.	5	3.8
Assoc. Prof.	20	15.2
Asst. Prof.	41	31.1
Lecturer, PhD	6	4.5
Res. Assist., PhD	6	4.5
Lecturer	31	23.5
Res. Assist.	23	17.4
Duration of working / year		
1–3 years	43	25.3
4–7 years	29	17.1
8–10 years	31	18.2
11 years or more	67	39.4
Administrative position status (*n* = 132)		
Yes	43	32.6
No	89	67.4
Weekly course load (Mean ± SD) (*n* = 132)	11.7 ± 11.98	
Time allocated to work at home weekly (hours)	7.74 ± 9.03	
Time allocated to housework at home daily (hours)	3.05 ± 4.37	
Time allocated to self daily (hours)	1.5 ± 1.82	
Time allocated to the spouse daily (hours)	1.15 ± 1.31	
Having children		
Yes	86	50.6
No	84	49.4
Time allocated to the child/children daily (hours)	1.5 ± 1.86	
Having elderly and/or disabled individuals who need help and support at home		
Yes	15	8.8
No	155	91.2
Person(s) helping with housework and childcare at home (*n* = 46)		
Family members (grandmother, great-grandmother, etc.)	24	52.2
Paid caregivers	22	47.8
Sharing domestic responsibilities with the spouse (*n* = 115)		
Sharing every day in the evening after work	62	53.9
Sharing on weekends	45	39.1
No sharing	8	7
Sharing childcare responsibilities with the spouse (*n* = 86)		
Sharing every day in the evening after work	61	70.9
Sharing on weekends	12	14
No sharing	13	15.1
Total	170	100

SD = standard deviation.

The mean scores of the university staff participating in the study were 15.40 ± 5.02 and 12.02 ± 4.76 on the Work–Family and Family–Work Conflict Scales, respectively. Academic staff had a higher score on the Work–Family and Family–Work Conflict Scales than administrative staff (*U* = 1942.5, *p* = 0.034; *U* = 1972, *p* = 0.042). The mean score of the Work–Family Conflict Scale is higher for the personnel who do not share domestic responsibilities with their spouses than for the personnel who share work every day in the evening and on weekends (χ^2^ = 8.855, *p* = 0.012). There is a statistically significant difference between the mean scores of the Family–Work Conflict Scale for those who have elderly and/or disabled individuals who need help and support at home compared with those who do not (U = 772, *p* = 0.030) and for those who have children compared to those who do not (*U* = 2917, *p* = 0.028). The mean rank of the Family–Work Conflict Scale scores of the personnel with extended family type is higher than the personnel with nuclear family type and single person (χ^2^ = 8.013, *p* = 0.018) (Table 2).

**Table 2.** Factors Affecting Participants’ Work–Family and Family–Work Conflict Scale Scores

	Work–Family Conflict Scale				Family–Work Conflict Scale			
	Mean rank	Q2	Q1–Q3	İstatistik	Mean rank	Q2	Q1–Q3	Statistics
Position								
Administrative staff	70.62	14	10–18.25	*U* = 1942.5 *Z* = −2.12 ***p* = 0.034**	71.39	10	9–11.5	*U* = 1972 *Z* = −2.032 ***p* = 0.042**
Academic staff	89.78	16	12–20		89.56	11	10–16	
						
Family type								
Nuclear family^a^	87.8	16	11–20	χ^2^ = 5.222 *p* = 0.073	84.10	10	10–15	χ^2^ = 8.013 ***p* = 0.018** **a–b; b–c**
Extended family^b^	75.6	14	10–20		123.25	16	11.5–17.75	
I live alone^c^	43.08	11	8.5–14.25		58.58	8	5–12.75	
Having children								
Yes	82.69	14	11–19.25	*U* = 3370 *Z* = −0.755 *p* = 0.451	93.58	11	10–16.25	*U* = 2917 *Z* = −2.195 ***p* = 0.028**
No	88.38	16	12–20		77.23	10	8.25–13	
Having elderly and/or disabled individuals who need help and support at home								
Yes	97.47	16	15–20	*U* = 983 *Z* = −0.989 *p* = 0.323	111.53	12	10–17	*U* = 772 *Z* = −2.174 ***p* = 0.030**
No	84.34	15	11–20		82.98	10	9–15	
Sharing domestic responsibilities with the spouse (*n* = 115)								
Sharing every day in the evening after work^a^	54.77	14	11–19	χ^2^ = 8.855 ***p* = 0.012** **c–a, b**	51.48	10	9–13	χ^2^ = 5.792 *p* = 0.055
Sharing on weekends^b^	56.47	14	11–19		64.36	11	10–18	
No sharing^c^	91.63	21	21–23.75		72.81	17.5	8.5–22.25	
Total Scale Score	15.40 ± 5.02				12.02 ± 4.76			

Q2 = Median; Q1 = 25. Quartile; Q3 = 75. Quartile; U = Mann–Whitney *U;* χ^2^ = Kruskal–Wallis H. ^a,b,c^ Indicate which groups there is a difference between. The bold text indicates a statistical difference, that is, *p* < 0.05.

A significant negative correlation was found between the Work–Family Conflict Scale and the daily time allocated to self and spouse (*r* = −0.163, *p* = 0.034; *r* = −0.189, *p* = 0.013). A significant positive correlation was found between the Family–Work Conflict Scale and the number of children (*r* = 0.185, *p* = 0.04), the age of the child (*r* = 0.204, *p* = 0.03), and the time allocated to the child or children per day (*r* = 0.250, *p* = 0.001) (Table 3).

**Table 3.** The Relationship Between the Work–Family and Family–Work Conflict Scales and Some Descriptive Variables

	Work–Family Conflict Scale		Family–Work Conflict Scale	
	*r*	*p*	*r*	*p*
Number of children	−0.034	0.719	0.185	**0.04**
Ages of children	−0.034	0.179	0.204	**0.03**
Time allocated to self daily (hours)	−0.163	**0.034**	−0.131	0.088
Time allocated to the spouse daily (hours)	−0.189	**0.013**	−0.109	0.157
Time allocated to the child/children daily (hours)	−0.088	0.256	0.250	**0.001**

The bold data indicates a statistical difference, that is, *p* < 0.05. *r* = Spearman’s correlation coefficient.

## Discussion

This study aimed to examine the level of work–family conflict and the factors affecting it among women academic and administrative staff. The level of work–family conflict among university staff was measured using the Work–Family Conflict Scale and the Family–Work Conflict Scale. The higher the score obtained from the scales, the higher the level of conflict.

Individuals try to maintain a balance between the roles and responsibilities required by work and family, and when the balance is not achieved, conflict between roles is experienced.^10,18^ In this study, work–family conflict was examined among female academic and administrative staff working in two different universities in a province in the Aegean Region of Turkey. In today’s world, especially, the pressure on women’s living conditions, work–family conflict, and the resulting stress negatively affect women’s physical, emotional, and mental health. The investigation of work–family conflict, which negatively affects women’s health, is becoming more and more important, especially in the female group.

In this study, the mean score of the Work–Family Conflict Scale (15.40 ± 5.02) was higher than the mean score of the Family–Work Conflict Scale (12.02 ± 4.76). Kahraman and Çelik, in a study conducted with academicians (*n* = 232) from two different universities in the same region, stated that academicians’ work–family conflicts were higher than family–work conflicts.^21^ Similar results in other studies in the literature draw attention to the fact that working women’s work–family conflict is higher than family–work conflict.^22,23^ This may be due to the fact that work responsibilities that need to be managed on a daily basis require a lot of time, concentration, and emotional management. It can also be attributed to reduced time and energy allocated to family life and self-care.^22^

In this study, having children, the number of children, and the age of the child/children were found to be related to family–work conflict. In his study, Özkan states that, among the administrative and academic staff of a university, those with three children experience more work–family conflict than those with two children.^24^ In a study conducted in China, it was reported that having only one child tends to reduce the level of family–work conflict by reducing family responsibilities and providing time to only one child.^25^ Likewise, there are studies reporting that family–work conflict is related to the number of children in the literature, while Kahraman and Çelik and Mustafayeva and Bayraktaroğlu reported that family–work conflict is not related to the number of children in their study results.^21,26^ This difference may result from the age of the child/children, as well as the number of children at work–family conflict. Because the higher the number of children in the age-group dependent on parents, the higher the level of work and family conflict.^6^ In a study examining work–family conflict in male and female academic staff, it was found that having children younger than 6 years and between 6 and 18 years of age affected work–family and family–work conflict.^27^ In a study conducted with 828 academic staff across Turkey, it was stated that the age of the youngest child requiring care was the determinant variable in family-work conflict. In other words, the need for care that decreases with the growth of the child is a factor that reduces family–work conflict.^28^

It was found that individuals with a large family type, who have elderly and/or disabled individuals in need of help and support at home, experience more family–work conflict. It is an expected situation that families with more people have more work responsibilities and carry more caregiver burden in terms of time and energy than families with fewer people. The family’s demand for the time and attention needed to take care of household chores, children, and the person in need of care is based on the size of the family, the composition of the family, and the number of family members who are dependent on other members.^29^ However, some study results reveal that work–family conflict does not show a significant difference according to whether there is a person in need of care at home.^24^

It was found that academic staff experienced more work–family and family–work conflict than administrative staff. In a similar study conducted in three universities in Norway, it was reported that academic staff experienced more work–family conflict than nonacademic staff.^30^ Carlson, Grzywack, and Kacmar argue that academic staff are more likely to experience work–family conflict than nonacademic staff, most of whom work during working hours, due to reasons such as flexible working hours and the need to allocate time for work at home.^31^

It was found that women who did not receive spousal support for their domestic roles experienced more work–family conflict. In the literature, there are results indicating that the support provided by the family and spouse and job sharing reduce the work–family conflict experienced by women.^6,32^ One of the factors affecting work–family conflict is workload. As the workload increases, work–family conflicts increase. In parallel with the decrease in workload, women are thought to be able to spend more time for themselves and their husbands and to be less likely to experience work–family conflicts. This is supported by the fact that women in our study experienced less work–family conflict as the time they allocated to themselves and their spouses increased.

It can be said that the level of work–family conflict among female academic and administrative staff is affected by variables associated with life. Work–family conflict, beyond creating a negative impact on family and work, leaves women’s health in a climate of silent threat in physical, emotional, and social terms.^15,33^

## Conclusion

The level of work–family conflict among the university staff participating in the study was found to be higher than the level of family–work conflict. There is a statistically significant difference between the Work–Family Conflict Scale and the status of sharing duties and domestic responsibilities with the spouse. A statistically significant difference was found between the Family–Work Conflict Scale and job, family type, having children, and having elderly and/or disabled individuals who need help and support at home. A significant negative correlation exists between the Work–Family Conflict Scale and the time allocated to oneself and their spouse on a daily basis. A significant positive correlation was found between the Family–Work Conflict Scale and the number of children, the age of the child, and the time allocated to the child/children per day. As stated in the literature on work–family conflict, the results of this study also revealed women’s need for support in work and family areas. It is thought that future studies in this field, addressing it in depth in large sample groups, will bring analytical approaches to it.

### Limitations of the study

The results of this study are limited to the female university staff who participated in the research. Another limitation of the study is that the physical, emotional, and mental health status of the participants was not questioned.

## Supplementary Material

Supplementary Data S1

## Abbreviations Used

χ^2^: Kruskal–Wallis H
Q1: 25. Quartile
Q2: Median
Q3: 75. Quartile
r: Spearman’s correlation coefficient
SD: standard deviation
SPSS: Statistical Package for Social Sciences
U: Mann–Whitney U
