# Supplementary material for: The Silent Threat in Women’s Health: Work and Family Conflict
Source: Womens Health Rep (New Rochelle). 2024 Sep 11;5(1):663–70. doi: 10.1089/whr.2024.0088 (PMC11462420; doi:10.1089/whr.2024.0088)
Supplement: Supplementary Data S1 [file whr.2024.0088_supp_datas1.pdf]

DATA COLLECTION FORM

Personal Information Form

1. Age:……………
2. Educational status

   a) High school graduate b) Bachelor's degree c) Master's degree d) Doctorate degree
3. Akademic title:

   a)Prof. b) Assoc. Prof. c) Asst. Prof. d) Lecturer, PhD e) Res. Assist., PhD

   f) Lecturer g) Res. Assist.
4. The unit where you work:……………………..…….
5. Duration of working / year as academic………..
6. Duration of working / year as administrative staff:…..…………….
7. Family type: a) Nuclear family b) Extended family c) I live alone
8. Income status:

   a) Low (Income less than expenditure) b) Medium (Income equal to expenditure)

   c) High (Income more than expenditure)
9. Marital status: a)Married b)Single
10. Duration of marriage / year:…………..
11. Age of spouse:……………...
12. Spouse's educational status

    a) High school graduate b) Bachelor's degree c) Master's degree d) Doctorate degree
13. Number of children:………
14. Ages of child/children:…….
15. Having administrative position status

    a)Yes b)No
16. Weekly course load:……….…..…..
17. Time allocated to work at home weekly (hours): ……………..
18. Time allocated to housework at home daily (hours): ……………..
19. Time allocated to self daily (hours): ……………
20. Time allocated to the spouse daily (hours):………….
21. Time allocated to the child/children daily (hours):………….…
22. Having elderly and/or disabled individuals who need help and support at home

    a)Yes b)No
23. Person(s) helping with housework and childcare at home

    a)Yes b)No
24. Sharing domestic responsibilities with the spouse

    a)Yes b)No

25. Sharing childcare responsibilities with the spouse

a)Yes b)No

	Strongly Disagree	Disagree	Undecided	Agree	Strongly Agree
**Work-Family Conflict Scale**					
1. The demands of my work interfere with my home and family life.					
2. The amount of time my job takes up makes it difficult to fulfill family responsibilities.					
3. Things I want to do at home do not get done because of the demands my job puts on me.					
4. My job produces strain that makes it difficult to fulfill family duties.					
5. Due to work-related duties, I have to make changes to my plans for family activities.					
**Family-Work Conflict Scale**					
1. The demands of my family or spouse/partner interfere with work-related activities.					
2. I have to put off doing things at work because of demands on my time at home.					
3. Things I want to do at work don't get done because of the demands of my family or spouse/partner.					
4. My home life interferes with my responsibilities at work such as getting to work on time, accomplishing daily tasks, and working overtime.					
5. Family-related strain interferes with my ability to perform job-related duties.					
